# The mechanism of colon tissue damage mediated by HIF-1α/NF-κB/STAT1 in high-altitude environment

**DOI:** 10.3389/fphys.2022.933659

**Published:** 2022-09-09

**Authors:** Junfei Cheng, Yuemei Sun, Jiaxin He, Zihan Wang, Wenbin Li, Rong Wang

**Affiliations:** ^1^ Lanzhou University School of Pharmacy, Lanzhou, Gansu, China; ^2^ The Logistics Support Force of Chinese People’s Liberation Army Pharmacy Department, Lanzhou, Gansu, China

**Keywords:** high altitude, colon, inflammation, hypoxia inducible factor-1α (HIF-1α), STAT1, NF-κB

## Abstract

The high-altitude environment damages the intestinal mucosal barrier, leading to a high incidence of intestinal diseases and seriously affects the working ability of people at high altitude. However, how high altitude induces intestinal mucosal barrier injury has not been well defined. The purpose of this study was to investigate the mechanism of colonic tissue injury induced by the influence of the high-altitude environment on the colonic microenvironment. Forty-eight SPF C57BL/6J mice were randomly divided into four groups: the control group and three other that were high-altitude exposure groups (Yushu, Qinghai; elevation: 4,010 m; 12 h, 24 h, 48 h). First, HE staining was used to observe the effect of the high-altitude environment on colon histomorphology of mice. The protein expression levels of claudin-1, occludin, and ZO-1 were analyzed by molecular biological methods. We found that altitude caused inflammatory damage to colon tissue. Intestinal hypoxia was measured with the hypoxic probe pimonidazole (PMDZ). Interestingly, we observed a decrease in the concentration of oxygen in the microenvironment in the colonic lumen. We sought to explore the mechanism of colonic mucosal barrier damage at different times when entering high altitude. The expression levels of hypoxia-inducible factors: HIF-1α, STAT1, and NF-κB and of inflammatory factors: IFN-γ, TNF-α, and IL-6 were significantly increased. This work highlights that the high-altitude environment leads to a reduction in the concentration of oxygen in the microenvironment of the colonic lumen, which disrupts the colonic mucosal barrier and ultimately induces and exacerbates intestinal injury.

## 1 Introduction

The intestinal mucosal barrier is an important part of the body’s defense and is reinforced by tight junction proteins, which effectively prevent harmful substances from entering the body (Chelakkot et al., 2018). Hypoxic environments can lead to inflammation of the small intestine (Shah, 2016), and it is also the focus of inflammatory bowel disease. In the colitis mouse model, the intestinal mucosa is in a state of intense hypoxia, suggesting that hypoxia is involved in colitis (Shah et al., 2008). The main threat to human health at high altitudes is hypoxia. The high incidence of diseases of the digestive system seriously affects the physical functioning and working ability of populations living in plateaus (Frühauf, 2014) and also affects the pharmacokinetic parameters of drugs (Zhang and Wang, 2022) such that the drugs cannot achieve effective therapeutic effects. However, the mechanism of colon inflammatory injury caused by the high-altitude environment is still unclear.

We previously screened for cytokines that may be involved in inflammatory injuries in high-altitude environments through protein chip technology (Wang et al., 2016). The results showed that compared with the control group, there were significant changes in several inflammatory factors in the serum of the rats in the high-altitude groups. Hypoxia-inducible factor 1α (HIF-1α), an O_2_-regulated protein, is a major transcription factor activated during hypoxia, and its stability is affected by the oxygen content. Studies have shown that hypoxic environment can lead to the activation of NF-κB in intestinal tissue (Nagpal and Yadav, 2017). NF-κB acts as a nuclear transcription factor and interferon regulator. After its activation, it enters the nucleus to promote the production of various inflammatory factors (Barnabei et al., 2021). NF-κB maintains the intestinal mucosal barrier. Signal transducer and activator of transcription 1 (STAT1) is an important member of a family of STAT proteins those act in a combinatorial fashion to help eliminate the expression of antibacterial and inflammatory response genes from foreign pathogens (Cohen Katsenelson et al., 2019). However, while inflammation is necessary to defend against foreign microbes, if not addressed in time, it can lead to cytokine overproduction, chronic inflammation, and even cancer (Fullerton and Gilroy, 2016). But the role of STAT1 in intestinal inflammation induced by high-altitude hypoxic environment has not been reported.

With the increase of high-altitude tourism and high-altitude workers, the incidence of intestinal-related diseases is high, and gastrointestinal reactions are one of the symptoms of acute altitude sickness (AMS). Gastrointestinal reactions caused by high-altitude environments mainly include nausea, vomiting, abdominal distension, diarrhea, etc. Among them, abdominal distention and diarrhea may be related to colon damage. It is urgent for us to further study the colonic inflammatory injury and related mechanisms caused by high-altitude environments. Since hypoxia plays an important role in regulating various physiological responses and pathological conditions, we hypothesized that decreased colonic tissue oxygen levels create hypoxic stress, which further exacerbates and accelerates inflammation and tissue damage. Here, we first need to clarify that the gut is a highly hypoxic environment, however, this “physiological hypoxia” has not been described in the high-altitude environment. In this study, relying on the established high-altitude field laboratory in Yushu, Qinghai (4,010 m), the morphological damage of the colon, colonic hypoxia, colonic barrier integrity, the expression of tight junction proteins, and the level of inflammatory signals in the colon were observed at different times of high-altitude hypoxia to evaluate inflammatory injury of colon tissue in the hypoxic environment. Molecular biology techniques were used to analyze the protein expression levels of HIF-1α, NF-κB, and STAT1 under high-altitude hypoxia environment. The mechanism of inflammatory injury of colon tissue provides a useful reference for the prevention and treatment of high-altitude intestinal diseases.

## 2 Materials and methods

### 2.1 Animals

All experiments were performed on 48 SPF-grade 8-week-old male C57BL/6J mice (body weight 20 ± 2 g, license number: SCXK (Beijing) 2019-0010), which were purchased from Beijing Speifu Biotechnology Co., Ltd.). All protocols were approved by the 940th Hospital of Joint Logistics Support Force of the Chinese People’s Liberation Army and were performed in accordance with relevant guidelines and regulations. The approval number is 2021KYLL175. The mice were placed under controlled temperature (20–25°C), humidity (50 ± 10%), and light (alternating 12-h light–dark cycle) conditions and fed following laboratory standards. Before the experiment started, they were adaptively reared in the SPF animal laboratory of the 940th Hospital of the Joint Logistics Support Force for 1 week.

### 2.2 Laboratory instruments and reagents

Microfuge 22R desktop refrigerated centrifuge (Beckman, Germany), ultra-low temperature refrigerator (Anhui Zhongke Duling Co., Ltd.), electric thermostatic water bath (Changzhou Putian Instrument Manufacturing Co., Ltd.), electronic analytical balance [Mettler-Toledo Instruments (Shanghai) Co., Ltd.], electric heating constant temperature blast drying oven (Shanghai Yuejin Medical Equipment Factory), physiological saline (Shijiazhuang Siyao Co., Ltd.), BSA24S-CW electronic analytical balance (Sartorius, Germany), paraformaldehyde fixative solution (Wuhan Sewell Biotechnology Co., Ltd.); TissueLyser-24 (Shanghai Jingxin Industrial Development Co., Ltd.), BCA protein concentration assay kit (Beijing Solarbio company), NE-PER™ Nuclear and Cytoplasmic Extraction Reagents (ThermoFisher Scientific, United States), Mouse IFN-γ enzyme-linked immunosorbent assay kit (Jianglai Biotechnology), Mouse TNF-α ELISA kit (Jianglai Biotechnology), Mouse IL-6 ELISA kit (Jianglai Biotechnology), Anti-Claudin 1 (Abcam, United States); Anti-Occludin (Abcam, United States), Anti-ZO1 (Abcam, United States); Anti-HIF-1α (Affinity Biosciences, United States); Anti-NF-κB p65 (Abcam, United States); Anti-STAT1 (Proteintech).

### 2.3 Experimental method

#### 2.3.1 Experimental animals and groups

We randomly divided healthy adult male SPF C57BL/6J mice into four groups, which were divided into the control group (C group, 1,500 m above sea level, Lanzhou), the high-altitude group for 12 h (H12 group, 4,010 m above sea level, Yushu, Qinghai), high-altitude group for 24 h (H24 group, 4010 m above sea level, Yushu, Qinghai), and high-altitude group for 48 h (H48 group, 4, 010 m above sea level, Yushu, Qinghai). The mice in the H24, H24, and H48 groups were transported by truck to the high altitude area and transported from Lanzhou to Qinghai within 12 h. After arriving at the destination, the experimental group were made to fast for 12 h before the experiment and the colon tissue collected at 12, 24, and 48 h. For the control group, the experiments were performed at Lanzhou.

#### 2.3.2 Colon tissue hematoxylin and eosin staining and pimonidazole staining

Six mice from C group and H12, H24 and H48 groups were taken and fixed, three of which were used for PMDZ staining and the other three for HE staining. Pimonidazole (60 mg/kg) was administered intraperitoneally at the corresponding time. The mice were killed by dislocation half an hour later. A 2-cm mouse colon tissue was dissected and fixed, washed with precooled normal saline to remove blood stains, dried with filter paper, and immediately placed in 4% paraformaldehyde for fixation. Dehydration, embedding, slicing, and staining with HE were performed after adequate fixation. Pathological changes and immunohistochemical experiments were conducted to study the effect of the high-altitude environment on the intestinal barrier.

The fixed tissue requiring immunohistochemistry was dehydrated, trimmed, and then embedded in paraffin, sectioned, dewaxed to water, quenched using endogenous catalase in the tissue, washed twice with buffer, and placed in a target retrieval reagent which was incubated at 90°C for 20 min for antigen retrieval. After cooling to room temperature, it was washed twice with buffer again. A protein-blocking agent was added to block the background staining. Incubate in FITC conjugated to mouse IgG1 monoclonal antibody (FITC-MAb1) primary antibody for 60 minutes at room temperature, washed twice with buffer, then incubate set with Rabbit anti-FITC conjugated with horseradish peroxidase secondary antibody for 30 min at room temperature, washed twice with buffer, and stop color development with DAB, counterstained, dehydrated in alcohol, clear, and mounted. A scanner was used to scan the fluorescence images in a panoramic view, and the fluorescence images of the three mice were collected in each group of colon tissues.

#### 2.3.3 Determination of inflammatory factors in colon tissue

Once the colon tissue was removed from the −80°C refrigerator, it was rinsed with pre-cooled PBS (0.01 M, pH = 7.4); the blood residue was removed, and 30–35 mg of colon tissue weighted. It was then placed in a corresponding volume of PBS (according to the ratio of 1:9). The colon tissue was ground in a prechilled homogenizer. Finally, the homogenate was centrifuged at 5,000 rpm for 10 min, and the supernatant was collected to detect the expression levels of IFN-γ, TNF-α, and IL-6 according to the instructions.

#### 2.3.4 FITC-dextran for the detection of intestinal permeability

Four groups of mice were made to fast for 12 h before the experiment, and FITC-dextran was dissolved in sterile water for injection. During the experiment, the mice were given FITC-dextran (500 mg/kg) by gavage, 4 h before the start of the experiment, and after 4 h of water deprivation, the blood samples were collected from the eye socket and the fluorescence value was detected by using a fluorescence microplate reader (excitation wavelength, 485 nm; emission wavelength, 520 nm), and quantitative analysis was performed according to the calibration curve of serum FITC-dextran concentration.

#### 2.3.5 Western blotting

A total of 45–55 mg of frozen intestinal tissue was taken in 1.5 ml of the EP viewer, 450–550 μL of the pre-prepared tissue lysis solution (high-efficiency RIPA lysis solution:PMSF = 100:1, V:V) was taken; two steel balls were added to each tube; and the colon tissue was placed in a pre-chilled homogenizer and homogenized three times of 1 min for each time. After homogenization, the cells were lysed on ice for 30 min, vortexed every 10 min, then centrifuged in a cryogenic centrifuge at 12,000 rpm for 10 min, and the supernatant was aspirated to get the total protein. After measuring the protein concentration with BCA, according to the ratio of supernatant to 4× loading buffer = 3:1 (V:V), it was mixed well, boiled at 100°C for 10 min to denature the protein, and then stored in aliquots at −80°C refrigerator.

A total of 15–40 mg of the tissue was cut into small pieces and placed in a microcentrifuge tube. The tissue was washed with PBS, centrifuged at 500 × *g* for 5 min, and the supernatant aspirated. The tissue was then homogenized using a tissue grinder in the appropriate volume of CER I. The volume ratio of CER I:CER II:NER reagents was maintained at 200:11:100 µL. The tube was vortexed vigorously on the highest setting for 15 s to fully suspend the cell pellet. It was intubated on ice for 10 min. Ice-cold CER II was added to the tube. The tube was vortexed for 5 s on the highest setting. It was intubated on ice for 1 min. The tube was then vortexed for 5 s on the highest setting and centrifuged for 5 min at maximum speed in a microcentrifuge (∼16,000 × *g*). The insoluble (pellet) fraction, which contains the nuclei, was suspended in ice-cold NER. The sample was placed on ice and vortexed for 15 s every 10 min, for a total of 40 min. The tube was centrifuged at maximum speed (∼16,000 × *g*) in a microcentrifuge for 10 min. The supernatant fraction was immediately transferred to a clean prechilled tube to get the nucleoprotein. This was placed on ice, and the extracts were stored at −80°C until use.

Equal amounts of proteins was loaded by 7–12% SDS-PAGE gel and transferred to polyvinylidene fluoride (PVDF) membranes. The membranes were blocked with 5% nonfat milk, and then incubated overnight with the antibodies against HIF-1α, STAT1, NF-κB P65, claudin-1, occludin, and ZO-1. The next day, the membranes were incubated with secondary antibodies, and protein bands were visualized by using ChemiDoc MP and quantified using Image Lab software.

#### 2.3.6 Statistical analysis

Data were processed using the SPSS 22.0 software. The data were expressed as mean ± standard deviation (SD). Multiple comparisons between the groups were conducted by one-way analysis of variance (ANOVA) followed by the LSD *post hoc* test. *p* < 0.05 was considered as significantly different.

## 3 Results

### 3.1 Effects of high altitude environment on the morphology of colonic mucosa in mice

**FIGURE 1:**
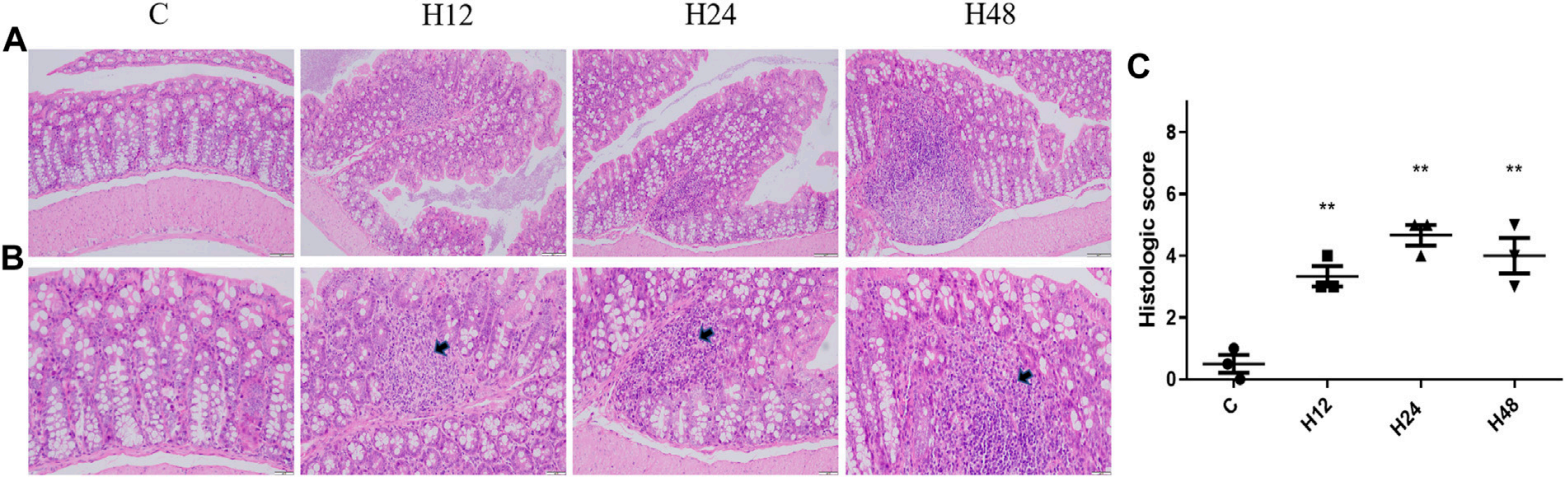
Changes in colonic barrier functions in mice from each group. **(A)** HE staining of colon tissue from mice in each group (×20). **(B)** HE staining of colon tissue from mice in each group (×40). **(C)** HE pathological scoring results. C, control group; H12 group, the high-altitude group for 12 h; H24 group, the high-altitude group for 24 h; H48, the high-altitude group for 48 h, *n* = 3. The data are presented as mean ± standard error of the mean. *p < 0.05 *vs*. N group, **p < 0.01 *vs*. C group.

The pathological results of the colon tissue of mice with high-altitude exposure at different times are shown in Figure 1. It can be seen that the colon tissue of the mice in the C group is normal in shape; the cells are closely arranged; and there is no obvious pathological change. High-altitude environments can lead to increased spacing between crypts in colon tissue, distortion of crypts, thickening of crypt bases, obvious shedding of mucosal epithelium, and disordered arrangement of intestinal glands. The results have shown that the high-altitude environment can lead to changes in colon cell morphology, infiltration of inflammatory cells, and destruction of the lamina propria, leading to damage of the intestinal mucosal barrier.

### 3.2 Effects of high altitude environment on the expression of tight junction protein in mouse colon tissue

**FIGURE 2:**
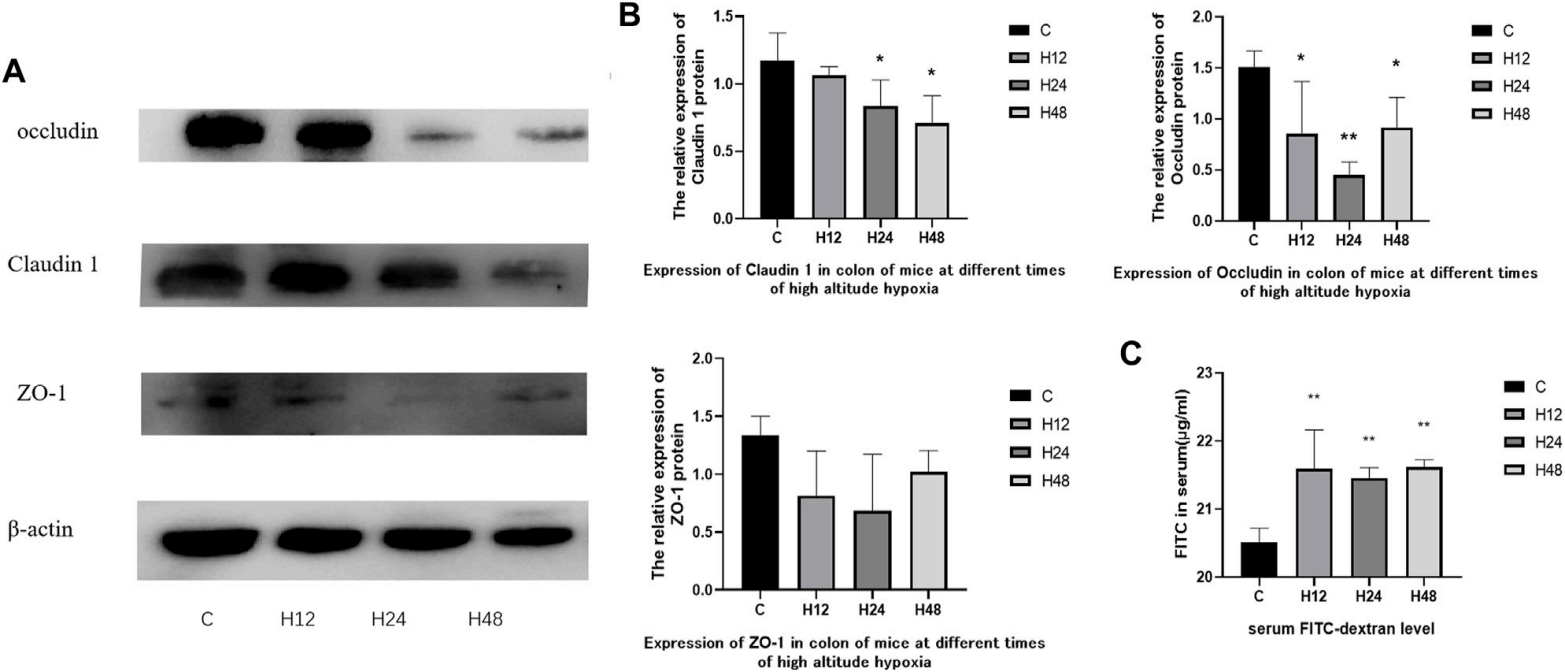
**(A,B)** Expression of tight junction protein in colon tissues of mice in each group, *n* = 3. **(C)** Serum FITC-dextran level, *n* = 5. C, control group; H12 group, the high-altitude group for 12 h; H24 group, the high-altitude group for 24 h; H48, the high-altitude group for 48 h. The data are presented as the mean ± standard error of the mean. *p < 0.05 *vs*. N group, **p < 0.01 *vs*. C group.

The experimental results of FITC-D4 and expression of tight junction proteins in mouse colon tissue is shown in Figure 2. The experimental results of FITC-D4 have shown that the high-altitude environment leads to an increase in intestinal permeability in the colon tissue of mice, indicating that the intestinal barrier is affected by the high-altitude environment. The expression of claudin-1 in mouse colon tissue was observed by hypoxia at different times, and the results have shown that the high-altitude environment leads to a decrease in claudin-1 expression in mouse colon tissue. Compared with the control group, the expression of claudin-1 protein in tissues was significantly downregulated by 9.1, 28.5, and 39.6%, respectively, after 12, 24, and 48 h of hypoxia at high altitude, while the tissue expression of occludin protein was significantly downregulated by 43.5, 69.9, and 39.3%, respectively. Tissue expression of ZO-1 protein showed a downward trend. The results of FITC-D4 and tight junction proteins have confirmed the results of HE staining from the level of the protein molecules that constitute the mechanical barrier, and the high-altitude exposure at different times destroyed the integrity of the colon tissue barrier in mice.

### 3.3 Effects of high altitude environment on colonic tissue hypoxia in mice

**FIGURE 3:**
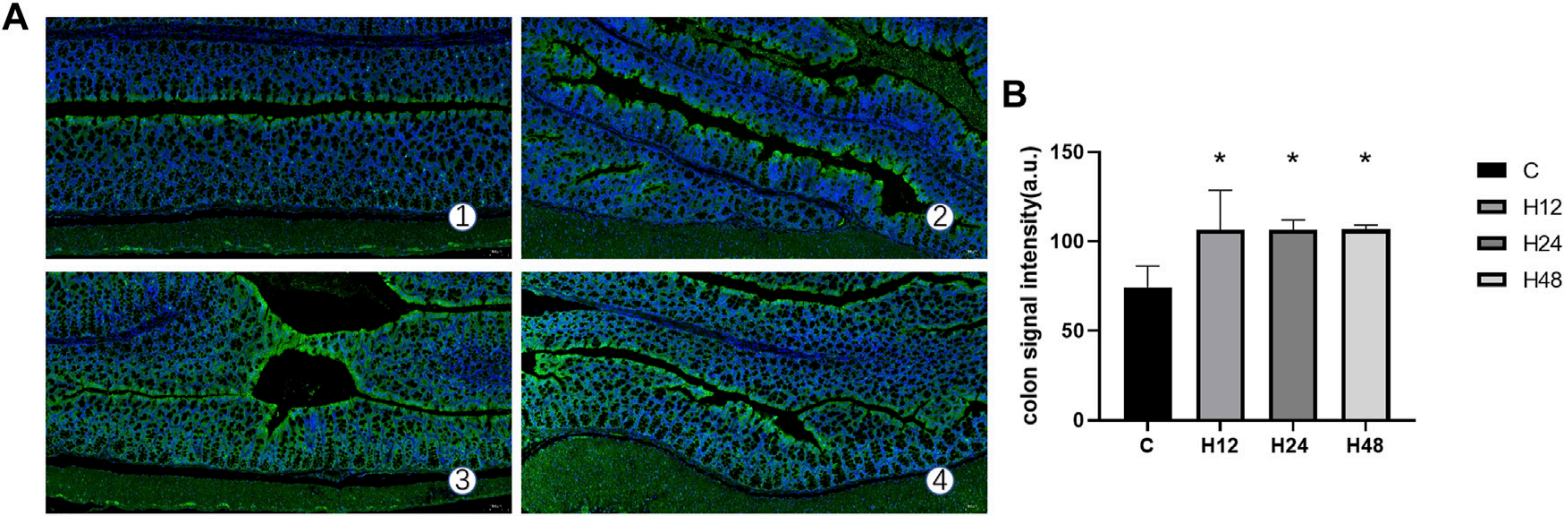
Hypoxia of colon tissue in each experimental group. **(A)** Expression of hypoxia probe signal intensity in colon tissue of each experimental group (10×), *n* = 3. **(B)** Quantitative experimental map of colon tissue hypoxia probe signal intensities in each experimental group, *n* = 3. 1: C, control group; 2: H12 group, the high-altitude group for 12 h; 3: H24 group, high-altitude group for 24 h; H48, high-altitude group for 48 h. The data are presented as the mean ± standard error of the mean. *p < 0.05 *vs*. N group, **p < 0.01 *vs*. C group.

The fluorescence signal intensity of the colon tissue hypoxia probe in each experimental group is shown in Figure 3. It can be seen from the data that when compared with the control group, the average fluorescence intensity of H12, H24, and H48 groups increased significantly by 43.19, 43.19, and 43.43%, respectively (*p* < 0.05). This shows that the hypoxic external environment such as high altitude can indeed lead to a decrease in oxygen concentration in the colon lumen of mice, which is likely to be the reason for the destruction of the integrity of the colonic mucosal barrier.

### 3.4 Effects of high altitude environment on inflammatory factors in mouse colon tissue

**FIGURE 4:**
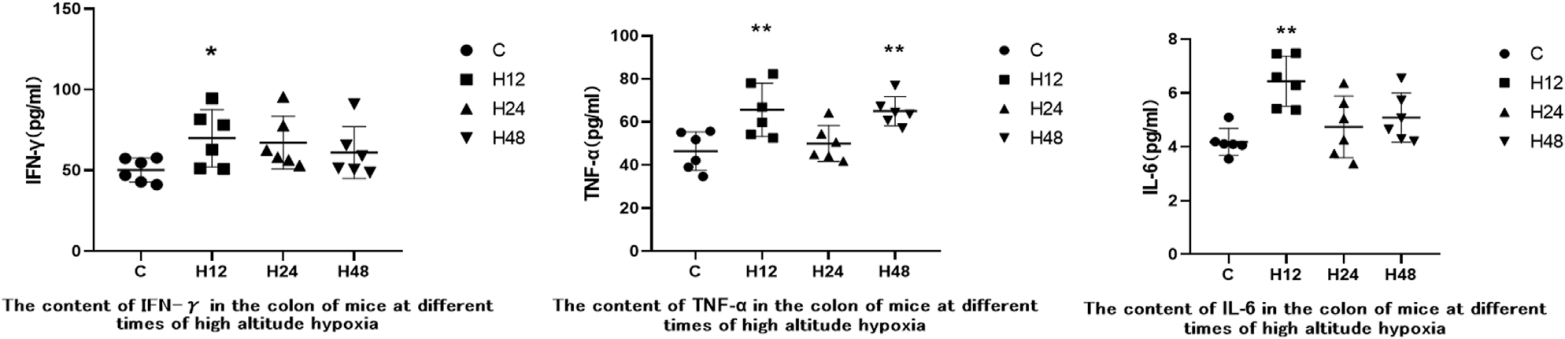
Expression of inflammatory factors in colon tissues of each experimental group. 1: C, control group; 2: H12 group, the high-altitude group for 12 h; 3: H24 group, high-altitude group for 24 h; H48, high-altitude group for 48 h, *n* = 6. The data are presented as the mean ± standard error of the mean. *p < 0.05 *vs*. N group, **p < 0.01 *vs*. C group.

ELISA kits were used to measure the expression of IFN-γ, TNF-α, and IL-6 in mouse colon tissue in order to observe inflammatory injury to mouse colon tissue caused by high-altitude environments. The results are shown in Figure 4. Entering the high-altitude environment results in varying degrees of increased inflammatory cytokines in mouse colon tissue. Compared with C group, H12 resulted in upregulation of IFN-γ, TNF-α, and IL-6 in mice colon tissue by 39.25, 41.36, and 53.78%, respectively. IFN-γ, TNF-α, and IL-6 were upregulated by 33.89, 7.68, and 13.14%, respectively, in the colon tissue of H24. IFN-γ, TNF-α, and IL-6 were upregulated by 21.55, 40.07, and 21.47%, respectively, in the colon tissue of H48. Compared with H12, inflammatory factors were downregulated at H24 and H48, but when compared with the C group, the inflammatory factors were still higher. Overall, the results of the elevated levels of the inflammatory factors indicated that the high-altitude environment caused some inflammatory damage to the colon tissue in mice.

### 3.5 Effects of high altitude environment on the expression of HIF-1α/STAT1/NF-κB protein in colon tissue of mice

**FIGURE 5:**
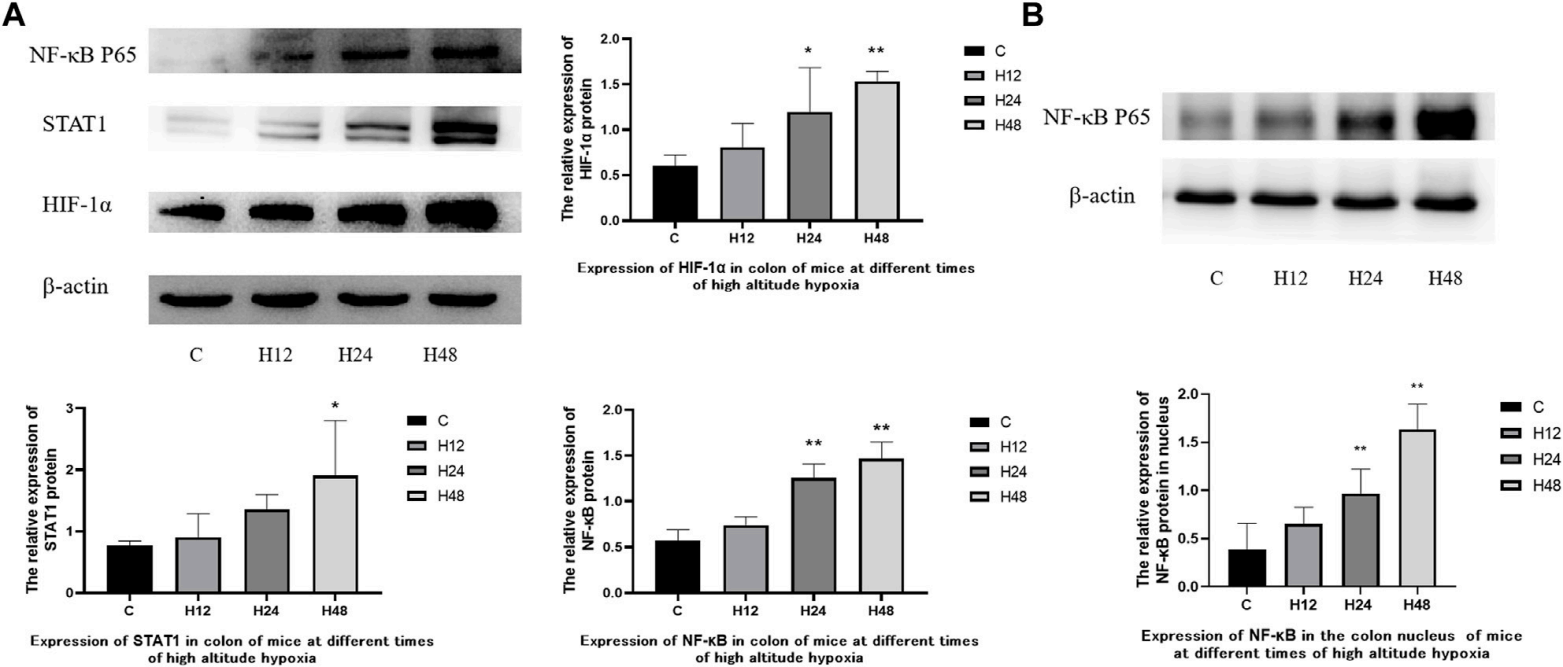
Expression of colon-associated proteins in mice at different times of high-altitude hypoxia. **(A)** Expression of related proteins in total protein. **(B)** Expression of NF-κB P65 in nuclear proteins. 1: C, control group; 2, H12 group, the high-altitude group for 12 h; 3, H24 group, the high-altitude group for 24 h; H48, high altitude group for 48 h, *n* = 6. The data are presented as the mean ± standard error of the mean. *p < 0.05 *vs*. N group, **p < 0.01 *vs*. C group.

The expression of various proteins in the colon tissue of mice on high-altitude exposure at different times was observed (Figure 5). The results showed that the high-altitude hypoxia environment led to an increase in the expressions of NF-κB, HIF-1α, and STAT1 in the colon tissue of mice. Compared with the C group, NF-κB, HIF-1α, and STAT1 were upregulated by 29.72, 33.97, and 18.48%, respectively, in the H12 group. NF-κB, HIF-1α, and STAT1 were upregulated by 119.74, 98.66 and 76.72%, respectively, in the H24 group. NF-κB, HIF-1α, and STAT1 were upregulated by 156.39, 154.99 and 148.59%, respectively, in the H48 group. At the same time, the expression of NF-κB in nuclear protein was upregulated by 67.9, 148.6 and 320.8% at 12, 24, and 48 h, respectively. The above results show that HIF-1α, NF-κB, and STAT1 were all upregulated and time dependent. HIF-1α acts as an oxygen concentration sensor to reflect hypoxia in the colon. Meanwhile, NF-κB and STAT1 reflect the activation of inflammation-related pathways in the colon. The results have indicated that colonic tissue damage may be mediated by the overexpression of HIF-1α/NF-κB/STAT1 under the high-altitude environment.

## 4 Discussion

AMS refers to high-altitude idiopathic disease at an altitude of more than 2,500 m. The most common clinical manifestations are headache, gastrointestinal symptoms, fatigue, dizziness, etc. AMS is caused by the hypoxic environment in the plateaus that seriously threatens the health and life safety of the people stationed in the plateaus, and significantly reduces the work efficiency of the working population. Among the symptoms of AMS, gastrointestinal (GI) reactions are the main symptoms (Zhang et al., 2018). Although it has been proved that the intestinal mucosal barrier is damaged at high altitudes, most of the research objects are small intestinal tissue. However, the mechanism of colonic tissue inflammatory injury mediated by low oxygen concentration in high-altitude environments is still unclear. The purpose of this study was to investigate the mechanism of inflammatory damage to colonic tissue caused by the oxygen concentration in the colonic lumen unraveling in the high-altitude environment. Our findings may provide effective measures for the search of prevention and treatment of intestinal diseases caused by high-altitude exposure.

In this study, we analyzed the pathological changes of the colon tissue in C57BL/6J mice after exposure to high-altitude hypoxia for 12, 24, and 48 h and found that the colon tissue in the high-altitude hypoxia group had obvious morphological changes, accompanied by inflammatory cell infiltration, indicating that high-altitude environments can lead to inflammatory damage in the colon tissue. The high-altitude environment induces increased colonic barrier permeability in mice at the overall animal level. Tight junctions between the intestinal epithelial cells are one of the important components of the intestinal mechanical barrier. Whereas, how high-altitude environments affect the tight junction proteins between colonic epithelial cells is unclear. Tight junction proteins are mainly composed of occludin-related proteins, claudin, and other proteins. The high-altitude environment reduces the protein expression levels of claudin-1, occludin, and ZO-1 in the colon tissue, suggesting that the colonic barrier function is impaired and colonic intestinal permeability has been increased. The gastrointestinal tract has unique oxygenation characteristics, and even in the normal physiological state, the intestinal mucosa epithelial cells are in a state of hypoxia, which is called “physiological hypoxia.” It is well known that the most severe characteristic of high-altitude environments is the reduced oxygen concentration. We used the hypoxic probe pimonidazole (PMDZ) to detect colonic hypoxia and quantified PMDZ retention, and the mean fluorescence intensity reflected the hypoxia probe signal intensity. We observed a significant increase in the fluorescence intensity of the colon tissue signal in the high-altitude groups, indicating that the high-altitude environment leads to aggravated “physiological hypoxia” in colon tissue.

**FIGURE 6:**
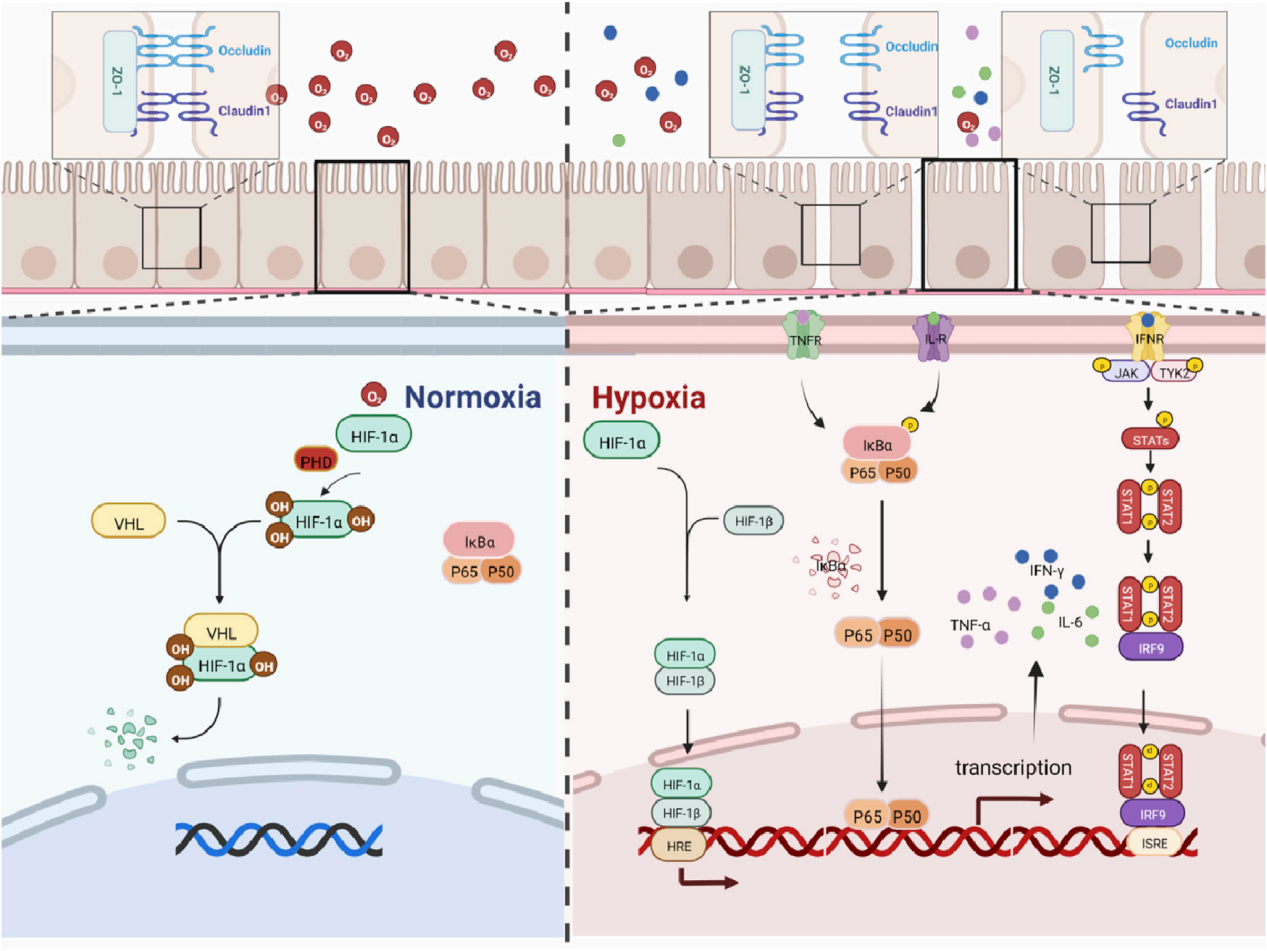
HIF-1α/NF-κB/STAT1 pathway map under high-altitude hypoxia.

HIF-1α, as an oxygen sensor, plays a key regulatory role in hypoxia-related diseases. In a hypoxic environment, HIF-1α is activated and regulates cell survival, metabolism, and tumorigenesis. Studies have shown that the activation of HIF-1α contributes to the progression of inflammatory bowel disease (Kim et al., 2018), while the inhibition of HIF-1α activity helps to alleviate intestinal inflammation (Kerber et al., 2020). The present findings have shown that the expression level of HIF-1α in mouse colon significantly increased in a time-dependent manner. Our findings suggest that high-altitude exposure induces an anoxic microenvironment in the colonic lumen and promotes inflammatory responses in the colon of mice, and the maximal activation of HIF requires the activation of the other pathways. NF-κB is an important nuclear transcriptional regulator that plays an important role in regulating inflammation, immune response, cell proliferation, transformation, apoptosis, tumorigenesis, and other cellular processes (Kunnumakkara et al., 2020). In addition, NF-κB transcriptional dysregulation leads to chronic inflammation and cell death (Barnabei et al., 2021). Studies have shown that by downregulating the expression of TLR4 and NF-κB, the body’s inflammatory response can be reduced (Ye et al., 2022), and it has a therapeutic effect on inflammatory bowel disease in the mouse model by inhibiting the over-activation of TLR4/NF-κB (Liu et al., 2017). Studies have shown that there is an NF-κB binding site in the HIF-1α gene promoter. This leads to an increase in the expression of HIF-1α mRNA (Bonello et al., 2007; van Uden et al., 2008). All the above studies have indicated that the activation of the NF-κB signaling is a key factor in causing intestinal inflammation. The current study shows that the expression of NF-κB in the colon of mice and the expression levels of inflammatory factors IFN-γ, TNF-α, and IL-6 in the colon tissue were significantly increased. IFN-γ, IL-6, and TNF-α are associated with increased IEC death and the disruption of gut epithelial barrier function in IBD (Neurath, 2014). At the same time, studies have shown that TNF-α and IFN-γ act synergistically to kill IEC cells through the STAT1 module (Woznicki et al., 2021). IL-6R signaling is known to induce phosphorylation of both STAT1 and STAT3 in T cells (Barnstorf et al., 2019). STAT proteins are well known for their roles in transducing cytokine-mediated signals and specifying Th cell differentiation. Among them, STAT1 is responsible for the transduction of type 1 IFN signals (α and β) and type 2 signals (γ) through a JAK1/2-dependent mechanism. In particular, it is a major signal transmitter of IFN-γ, an important mediator of immune responses and inflammation (Lee et al., 2021). Accumulating evidence have indicated that STAT1 is also involved in the regulation of HIF-1α, and studies have shown that STAT1 can activate HIF-1α (Chen et al., 2019). At the same time, the more classic pathway between NF-κB and STAT1 is NF-κB/JAK/STAT1, and studies have shown that multiple inflammatory factors can play a role through the NF-κB-JAK/STAT1 pathway, such as IL-6R, IL-3R, and IFN receptor (IFN-R) (Coskun et al., 2013). The current findings suggest that the expressions of STAT1, NF-κB, and HIF-1α proteins are hyper-activated with increasing high low-oxygen exposure time, which ultimately leads to inflammatory damage in the colon in mice. The relationship between HIF-1α/NF-κB/STAT1 has been reported in the literature, but the mechanism of colonic barrier damage caused by the high-altitude environment has not been reported, so we studied it. The abovementioned results further provide evidence for the conclusion that high low oxygen exposure induces hyperactivation of HIF-1α/NF-κB/STAT1 signaling in mouse colon tissue, resulting in colonic inflammatory damage. It has been suggested that high low-oxygen exposure causes intestinal inflammation, and the longer the exposure time, the more severe the damage to the colonic barrier in mice. The mechanism is shown in Figure 6.

In conclusion, high altitude significantly induces the reduction of oxygen concentration in the colonic microenvironment. This change in the microenvironment results in time-dependent damage to the colonic barrier in mice. At the same time, high-altitude exposure significantly induces the activation of inflammatory signaling in mice colon. However, this is an animal model study, and we will collect more evidence in the clinic in the future. In a word, our findings suggest that the HIF-1α/NF-κB/STAT1 pathway plays an important role in inflammatory colon injury.

## Data Availability

The original contributions presented in the study are included in the article; further inquiries can be directed to the corresponding authors.

## References

[B1] Barnabei L. Laplantine E. Mbongo W. Rieux-Laucat F. Weil R. (2021). NF-κB: At the borders of autoimmunity and inflammation. Front. Immunol. 12, 716469. 10.3389/fimmu.2021.716469 34434197 PMC8381650

[B2] Barnstorf I. Borsa M. Baumann N. Pallmer K. Yermanos A. Joller N. (2019). Chronic virus infection compromises memory bystander T cell function in an IL-6/STAT1-dependent manner. J. Exp. Med. 216 (3), 571–586. 10.1084/jem.20181589 30745322 PMC6400541

[B3] Bonello S. Zähringer C. BelAiba R. S. Djordjevic T. Hess J. Michiels C. (2007). Reactive oxygen species activate the HIF-1alpha promoter via a functional NFkappaB site. Arterioscler. Thromb. Vasc. Biol. 27 (4), 755–761. 10.1161/01.ATV.0000258979.92828.bc 17272744

[B4] Chelakkot C. Ghim J. Ryu S. H. (2018). Mechanisms regulating intestinal barrier integrity and its pathological implications. Exp. Mol. Med. 50 (8), 103–109. 10.1038/s12276-018-0126-x PMC6095905 30115904

[B5] Chen Z. Mou L. Pan Y. Feng C. Zhang J. Li J. (2019). CXCL8 promotes glioma progression by activating the JAK/STAT1/HIF-1α/Snail signaling Axis. Onco. Targets. Ther. 12, 8125–8138. 10.2147/OTT.S224721 31686858 PMC6783399

[B6] Cohen Katsenelson K. Stender J. D. Kawashima A. T. Lordén G. Uchiyama S. Nizet V. (2019). PHLPP1 counter-regulates STAT1-mediated inflammatory signaling. Elife 8, e48609. 10.7554/eLife.48609 31408005 PMC6692130

[B7] Coskun M. Salem M. Pedersen J. Nielsen O. H. (2013). Involvement of JAK/STAT signaling in the pathogenesis of inflammatory bowel disease. Pharmacol. Res. 76, 1–8. 10.1016/j.phrs.2013.06.007 23827161

[B8] Frühauf H. (2014). Every mountain too high when nausea strikes: gastrointestinal function at high altitude. Praxis 103 (14), 825–832. German. 10.1024/1661-8157/a001718 24985228

[B9] Fullerton J. N. Gilroy D. W. (2016). Resolution of inflammation: a new therapeutic frontier. Nat. Rev. Drug Discov. 15 (8), 551–567. 10.1038/nrd.2016.39 27020098

[B10] Kerber E. L. Padberg C. Koll N. Schuetzhold V. Fandrey J. Winning S. (2020). The importance of hypoxia-inducible factors (HIF-1 and HIF-2) for the pathophysiology of inflammatory bowel disease. Int. J. Mol. Sci. 21 (22), 8551. 10.3390/ijms21228551 PMC7697655 33202783

[B11] Kim Y. E. Lee M. Gu H. Kim J. Jeong S. Yeo S. (2018). HIF-1α activation in myeloid cells accelerates dextran sodium sulfate-induced colitis progression in mice. Dis. Model. Mech. 11 (7), dmm033241. 10.1242/dmm.033241 29967068 PMC6078398

[B12] Kunnumakkara A. B. Shabnam B. Girisa S. Harsha C. Banik K. Devi T. B. (2020). Inflammation, NF-κB, and chronic diseases: How are they linked? Crit. Rev. Immunol. 40 (1), 1–39. 10.1615/CritRevImmunol.2020033210 32421977

[B13] Lee H. Lee D. H. Oh J. H. Chung J. H. (2021). Skullcapflavone II suppresses TNF-α/IFN-γ-Induced TARC, MDC, and CTSS production in HaCaT cells. Int. J. Mol. Sci. 22 (12), 6428. 10.3390/ijms22126428 34208434 PMC8233710

[B14] Liu J. Chen Y. Liu D. Liu W. Hu S. Zhou N. (2017). Ectopic expression of SIGIRR in the colon ameliorates colitis in mice by downregulating TLR4/NF-κB overactivation. Immunol. Lett. 183, 52–61. 10.1016/j.imlet.2017.01.015 28153604

[B15] Nagpal R. Yadav H. (2017). Bacterial translocation from the gut to the distant organs: An overview. Ann. Nutr. Metab. 71 (1), 11–16. 10.1159/000479918 28950279

[B16] Neurath M. F. (2014). Cytokines in inflammatory bowel disease. Nat. Rev. Immunol. 14 (5), 329–342. 10.1038/nri3661 24751956

[B17] Shah Y. M. Ito S. Morimura K. Chen C. Yim S. H. Haase V. H. (2008). Hypoxia-inducible factor augments experimental colitis through an MIF-dependent inflammatory signaling cascade. Gastroenterology 134 (7), 2036–2048. 2048.e1-3. 10.1053/j.gastro.2008.03.009 18439915 PMC2533811

[B18] Shah Y. M. (2016). The role of hypoxia in intestinal inflammation. Mol. Cell. Pediatr. 3 (1), 1. 10.1186/s40348-016-0030-1 26812949 PMC4728161

[B19] van Uden P. Kenneth N. S. Rocha S. (2008). Regulation of hypoxia-inducible factor-1alpha by NF-kappaB. Biochem. J. 412 (3), 477–484. 10.1042/BJ20080476 18393939 PMC2474706

[B20] Wang C. Wang R. Xie H. Sun Y. Tao R. Liu W. (2016). Effect of acetazolamide on cytokines in rats exposed to high altitude. Cytokine 83, 110–117. 10.1016/j.cyto.2016.04.003 27104804

[B21] Woznicki J. A. Saini N. Flood P. Rajaram S. Lee C. M. Stamou P. (2021). TNF-α synergises with IFN-γ to induce caspase-8-JAK1/2-STAT1-dependent death of intestinal epithelial cells. Cell Death Dis. 12 (10), 864. 10.1038/s41419-021-04151-3 34556638 PMC8459343

[B22] Ye Y. Warusawitharana H. Zhao H. Liu Z. Li B. Wu Y. (2022). Tea polyphenols attenuates inflammation via reducing lipopolysaccharides level and inhibiting TLR4/NF-κB pathway in obese mice. Plant Foods Hum. Nutr. 77, 105–111. 10.1007/s11130-021-00937-0 35138518

[B23] Zhang J. Wang R. (2022). Changes in CYP3A4 enzyme expression and biochemical markers under acute hypoxia affect the pharmacokinetics of sildenafil. Front. Physiol. 13, 755769. 10.3389/fphys.2022.755769 35153825 PMC8829446

[B24] Zhang W. Jiao L. Liu R. Zhang Y. Ji Q. Zhang H. (2018). The effect of exposure to high altitude and low oxygen on intestinal microbial communities in mice. PLoS One 13 (9), e0203701. 10.1371/journal.pone.0203701 30208088 PMC6135514

